# Concurrent Evolution of Biomechanical and Physiological Parameters With Running-Induced Acute Fatigue

**DOI:** 10.3389/fphys.2022.814172

**Published:** 2022-02-11

**Authors:** Gäelle Prigent, Salil Apte, Anisoara Paraschiv-Ionescu, Cyril Besson, Vincent Gremeaux, Kamiar Aminian

**Affiliations:** ^1^Laboratory of Movement Analysis and Measurement, École Polytechnique Fédérale de Lausanne (EPFL), Lausanne, Switzerland; ^2^Sport Medicine Unit, Division of Physical Medicine and Rehabilitation, Swiss Olympic Medical Center, Lausanne University Hospital, Lausanne, Switzerland; ^3^Institute of Sport Sciences, University of Lausanne, Lausanne, Switzerland

**Keywords:** running, biomechanics, physiology, perceived fatigue, wearable sensors

## Abstract

Understanding the influence of running-induced acute fatigue on the homeostasis of the body is essential to mitigate the adverse effects and optimize positive adaptations to training. Fatigue is a multifactorial phenomenon, which influences biomechanical, physiological, and psychological facets. This work aimed to assess the evolution of these three facets with acute fatigue during a half-marathon. 13 recreational runners were equipped with one inertial measurement unit (IMU) on each foot, one combined global navigation satellite system-IMU-electrocardiogram sensor on the chest, and an Android smartphone equipped with an audio recording application. Spatio-temporal parameters for the running gait, along with the heart rate, its variability and complexity were computed using validated algorithms. Perceived fatigability was assessed using the rating-of-fatigue (ROF) scale at every 10 min of the race. The data was split into eight equal segments, corresponding to at least one ROF value per segment, and only level running parts were retained for analysis. During the race, contact time, duty factor, and trunk anteroposterior acceleration increased, and the foot strike angle and vertical stiffness decreased significantly. Heart rate showed a progressive increase, while the metrics for heart rate variability and complexity decreased during the race. The biomechanical parameters showed a significant alteration even with a small change in perceived fatigue, whereas the heart rate dynamics altered at higher changes. When divided into two groups, the slower runners presented a higher change in heart rate dynamics throughout the race than the faster runners; they both showed similar trends for the gait parameters. When tested for linear and non-linear correlations, heart rate had the highest association with biomechanical parameters, while the trunk anteroposterior acceleration had the lowest association with heart rate dynamics. These results indicate the ability of faster runners to better judge their physiological limits and hint toward a higher sensitivity of perceived fatigue to neuromuscular changes in the running gait. This study highlights measurable influences of acute fatigue, which can be studied only through concurrent measurement of biomechanical, physiological, and psychological facets of running in real-world conditions.

## Introduction

The tremendous increase in the popularity of running (Rothschild, 2012) as a sport has hastened the need to understand the risk factors for running related injuries (RRI) arising out of maladaptation to training. While the direct relation of biomechanical risk factors and training load to the instances of lower extremity RRIs is debated (Ceyssens et al., 2019; Fredette et al., 2021), these factors are understood to be influenced by acute fatigue, especially resulting from endurance running (Verschueren et al., 2020). In this context, acute fatigue can be understood as the decline in performance caused by physical exertion during sports (Knicker et al., 2011), measured during or immediately after the sporting activity. Fatigue can be characterized as the inability to maintain the intensity of a sub-maximal exercise, caused by the change in the underlying interdependence between the central drive from the motor cortex and the contractile function of the muscles (Vargas and Marino, 2014; Enoka and Duchateau, 2016). Since fatigue depends on the interactions between performance and perceived fatigability, direct measurement of fatigue is difficult (Enoka and Duchateau, 2016). It is often investigated by measuring its concomitant effects on cardiovascular, neuromuscular, and psychological states via sensor-based approaches and self-reported scores on questionnaires (Thorpe et al., 2017). Other approaches include blood tests for lactate, cortisol, etc. and performance monitoring on functional tests like countermovement jump and maximum voluntary contraction (Bourdon et al., 2017). However, these two modalities are constrained to endpoint measurements and thus only useful for testing pre-to-post responses.

The influence of fatigue on autonomic cardiac control can be estimated through the dynamics of heart rate (Schmitt et al., 2015), such as heart rate variability (HRV) and heart rate complexity (HRC). These dynamics are computed from the electrocardiogram (ECG) signal obtained from wearable belts with single-lead electrodes or multi-lead stationary heart rate monitors (Billman et al., 2015); breathing rate can be measured using gas exchange systems in lab, or in-field with wearable strain sensors (Jayasekera et al., 2021). Acute fatigue can lead to a drop in the HRV metrics such as root mean square of successive differences between normal heartbeats (*RMSSD*) and the standard deviation of the heartbeat intervals (*SDNN*) when measured during exercise (Casties et al., 2006; Gronwald et al., 2020a). HRC variables such as the detrended fluctuation analysis (*DFA-α1*) coefficient, quantifying the degree of correlation of time series, respond to organismic demands during high intensity exercises (Hautala et al., 2003; Gronwald et al., 2021). Since running biomechanical parameters such as contact time, flight time, trunk flexion angle, vertical stiffness, ground reaction forces (GRF), etc. change in response to acute fatigue (Apte et al., 2021), continuous monitoring of these parameters can assist in understanding the effect of fatigue on neuromuscular function (Paquette et al., 2020). While gait spatiotemporal parameters, body segment kinematics, and GRF can be measured directly, vertical stiffness is typically estimated by modeling the running gait as a spring-mass model (Blickhan, 1989; Morin et al., 2005). The runner is considered as a point mass and the supporting leg as a linear spring, with the vertical stiffness characterizing the motion of the center of mass (COM) in response to the vertical GRF. Running biomechanics can be measured through stationary systems such as motion capture systems, force plates, and video camera and/or via wearable sensors such inertial measurement units (IMU), insoles with embedded pressure sensors, and global navigation satellite system (GNSS) receivers (Novacheck, 1998; Benson et al., 2018).

The number of studies on continuous and field monitoring of running-induced acute fatigue remains scarce, despite the recent proliferation of wearable measurement systems and movement analysis algorithms in sports science (Camomilla et al., 2018; Apte et al., 2021). Within these, some studies focused on the classification of fatigued and non-fatigued states using machine learning techniques based on statistical features or composite indices (Eskofier et al., 2012; Buckley et al., 2017; Op De Beéck et al., 2018; Clermont et al., 2019), which preclude the investigation of interpretable biomechanical or cardiovascular parameters. Studies examining the response of individual biomechanical parameters during long-distance running (≥10 km) have predominantly analyzed the parameter values at different distances (Alfuth and Rosenbaum, 2011; Strohrmann et al., 2012; Ruder et al., 2019; Meyer et al., 2021). This approach has an implicit assumption that different participants develop similar levels of fatigue at similar distances during the run, which may not be true for a heterogeneous participant group employing a variety of pacing strategies. Similarly, existing research on the continuous monitoring of heart rate dynamics and cardiac drift (Billat et al., 2019; Gronwald and Hoos, 2020; Gronwald et al., 2021) has generally considered their evolution over the distance of the run. Combined together, these studies investigate the neuromuscular and cardiovascular response to acute fatigue, but not their concurrent evolution and association. Neither do they assess the perceived fatigability and thus the psychological states during the run. Due to the complex nature of fatigue, perceived fatigability and the association between neuromuscular and cardiovascular response can provide a better global overview from a complex system perspective rather than a single biomechanical or physiological parameter (Venhorst et al., 2018; Balagué et al., 2020). Thus, rating of perceived exertion (RPE) (Borg, 1982) or rating of fatigue (ROF) (Micklewright et al., 2017) can provide a more holistic idea of central regulation, especially during the context of an actual running race that involves pacing strategies, making their investigation pertinent (Millet, 2011; Pageaux and Lepers, 2016).

In this work, we aimed to investigate the concurrent evolution of running biomechanics and heart rate dynamics in response to perceived fatigability for recreational runners, using body-worn smartphone, IMU, GNSS and ECG sensors. Furthermore, we computed the association between the two set of parameters and studied its evolution with perceived fatigability. Hereafter, perceived fatigability is alternately referred to as ROF and/or fatigue, since it is a reference for acute fatigue.

## Materials and Equipment

**FIGURE 1:**
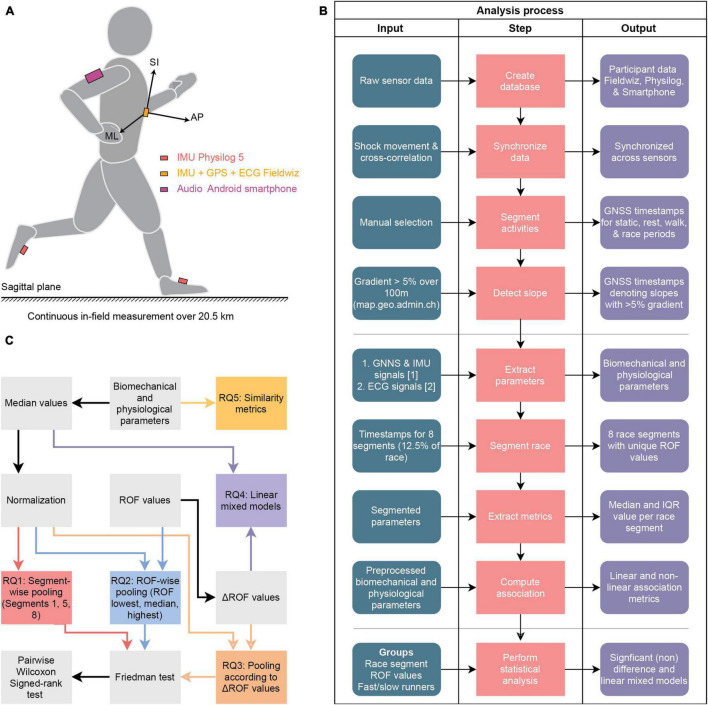
Sensor setup and data analysis process, **(A)** sensor configuration used for the measurement, where AP, SI, and ML denote the anterior-posterior, the superior-inferior, and the medio-lateral axis **(B)** flowchart for the overall procedure, showing three blocks for the pre-processing, feature extraction, and statistical analysis **(C)** statistical analysis procedure where the biomechanical and physiological parameters generated in **(B)** and the recorded ROF values are used as inputs. ROF, Rating-of fatigue; ECG, electrocardiogram; IMU, inertial measurement unit; GNSS, global navigation satellite system; IQR, interquartile range; RQ, research question.

We conducted measurements with 13 healthy participants, six (4 males, 2 females, age: 35.5 ± 9.3 y.o.) during the Lausanne half-marathon (Switzerland, 27th Oct. 2019) and seven (7 males age: 35.6 ± 5.8 y.o.) during a 21.5 km race-simulation run in Rif (Salzburg, Austria, 25–29th November 2020). The race-simulation in Rif was organized because of race cancelations in 2020 due to the pandemic situation. The half-marathon was chosen in order to avoid the walking periods that inexperienced participants can have during a full marathon, as we observed during pilot studies. EPFL human research ethics committee (HREC 039-2018) approved the study and all participants provided written consent before the data collection. As shown in Figure 1A, participants were equipped with a GNSS-IMU-ECG sensor (*Fieldwiz, ASI, Switzerland)* on the chest using a belt with electrodes (*Polar Pro Strap, Polar Electro Oy, Finland*), an IMU sensor (*Physilog 5, Gaitup SA, Switzerland*) on each feet, and an Android smartphone on the upper arm. Apart from the sensor setup, the participants dressed as they would for an endurance running race. Following their personal warm-up, the participants were equipped with the sensor setup and were instructed to give their best during the run.

The *Fieldwiz* and *Physilog 5* wearable sensors were chosen because they have already been used successfully for continuous analysis of running in the field (Apte et al., 2020; Meyer et al., 2021). *Fieldwiz* was used with a sampling frequency of 200 Hz for the IMU, 250 Hz for the ECG, and 10 Hz for the GNSS receiver. The *Physilog 5* IMU was sampled at 512 Hz, with a range of ± 16 g m/s^2^ for the accelerometer and ± 2,000 deg/s for the gyroscope. We installed a custom-built application on the smartphone, which reminded the wearer to speak out their rating of fatigue (ROF) on a scale of 1–10 (Micklewright et al., 2017) and recorded this audio with a timestamp. We configured the application to create a reminder every 10 min and subsequently record for a period of 30 s. The audio files were manually transcribed to store the recorded ROF value.

## Methods

The flowchart of the overall procedure for the pre-processing, feature extraction, and statistical analysis is presented in Figure 1B, and detailed explanations are provided in the sections “Preprocessing,” “Feature Extraction,” and “Statistical Analysis” respectively. In addition, Figure 1C provides detailed information about the statistical analysis.

### Preprocessing

The pre-processing steps include database organization, synchronization of the sensors, segmentation of the different activities (static pre/post, resting pre/post, walking pre/post, and race periods), and slope detection (Figure 1B). A shock movement, which consists of a fast up and down movement on the vertical axis while holding all sensors together, was performed before and after the race for synchronizing the *Fieldwiz* and *Physilog 5* wearable sensors (Caruso et al., 2019). As the same motion was recorded on the accelerometer of both sensors, we could compute the lag between the acceleration signal of both sensors using cross-correlation; this lag was then used to adjust their timestamps. We decided to restrain the analysis to the bouts of level running to avoid any biomechanical and physiological changes biased by inclined running. We used the official mapping platform of the Swiss Confederation *(map.geo.admin.ch)* to detect slopes on the Lausanne marathon route and computed the distance using the Haversine formula (Robusto, 1957) with the latitude and longitude information from the GNSS sensor. Slopes were defined as race segments having a gradient greater than 5% over 100 meters and corresponding race sections were excluded from the data. To avoid this procedure, we selected a relatively flat course for the run in Rif, with all gradients below the 5% level.

### Feature Extraction

#### Biomechanical Parameters

The accelerometer, gyroscope, and speed signals from the *Fieldwiz* sensor were processed to remove outliers that were more than two standard deviations away from the mean value over a race segment window and replaced with linearly interpolated values. To investigate the orientation of the trunk and its evolution throughout the race, we computed two additional metrics –*a*_*AP*_: the ratio of the acceleration along the anterior-posterior direction and the running speed (*v*) and *a*_*ML*_: the ratio of the acceleration along the medio-lateral direction and the running speed. Normalization with speed was carried out to investigate the response to fatigue and not the secondary effects of the change in speed. Using validated algorithms (Falbriard et al., 2018, 2020), the raw signals from the foot IMUs were initially used to divide the race into gait cycles based on mid-swings. Following this, we estimated the temporal parameters such as contact time (*t_c_*), flight time (*t_f_*), swing time (*t_s_*), and cycle time (*t_g_*), and kinematics parameters like peak swing velocity of the foot (ω_*s*_), foot strike angle in sagittal plane (*FSA*), and foot eversion angle (*FEA*) at initial contact. We obtained one value of each spatiotemporal parameter per gait cycle for the right and the left foot, but we used only the information from the right foot for all participants for the subsequent analysis and removed the first and last 10 steps of the race to avoid any transient effects. To understand the storage and return of elastic energy, we computed the vertical stiffness (*k*_*vert*_), using the spring mass model to characterize running (Morin et al., 2005). To consider the positive and negative work during running, we investigated the duty factor of the gait (Alexander, 1991) defined as the ratio between contact and stride time. The computation of the above-mentioned parameters are explained in the publication from Meyer et al. (2021).

#### Physiological Parameters

The physiological parameters were extracted from the raw ECG signal in four main steps: (i) data segmentation, (ii) QRS-complexes detection, (iii) R-peak correction, and (iv) feature extraction. Following the recommendation of Task Force of the European Society of Cardiology the North American Society of Pacing Electrophysiology (1996), the raw ECG signal was segmented into 120 s rolling windows with a 110 s overlap, thus allowing an adequate time resolution with a parameter computation every 10 s. On each window, the signal was resampled from 250 to 1,000 Hz using linear interpolation (*“interp1”* MATLAB function) to improve the robustness of R-wave detection. Then, a non-linear filtering method was used for QRS-complexes detection, which creates a coefficient vector based on short- and long-term energies in the signal and multiplies it with the signal itself to heighten peaks and suppress perturbations (Yazdani et al., 2018). Further, an R-peak correction procedure was applied to address the artifacts caused by electrode movements and poor skin-electrode contact. RR interval values greater than three times the standard deviation around the median of the 60 neighboring RR intervals were considered as outliers (Giles and Draper, 2018; Rincon Soler et al., 2018) and replaced using spline interpolation (Tarvainen et al., 2014). Finally, segments with artifact rates < 5% were considered valid for further heart rate variability (HRV) analyses (Rogers et al., 2021). Time-domain HRV metrics included beats per minute (*BPM*), *SDNN*, and *RMSSD*. Following this, the cardiac cost (*CC*) i.e., the ratio between *BPM* and running speed, was computed (Billat et al., 2020). Frequency domain analysis covered the low (0.04−0.15 Hz—LF) and high (0.04−1.5 Hz—HF) frequency components, as well as the ratio between the two bands (*LF/HF* ratio). The *HF* band was extended to 1.5 Hz to cover respiratory frequency during running (Casties et al., 2006; Cottin et al., 2007), making this metric specific to exercise assessment and not comparable to reference values at rest. *HF* and *LF* powers were normalized to the total variance in order to reveal contributions from the different spectral components. For the non-linear methods, we extracted the quantitative indices of the Poincare plot, represented by the transverse (*SD1*) and longitudinal (*SD2*) axes of the ellipsis (Kumar et al., 2017). To characterize the fractal correlation properties of HR time, we investigated the short-term (*DFA-α_1_*; window width: 4 < *n* < 16 beats) and long-term (*DFA-α_2_*; window width: 16 < *n* < 64 beats) scaling exponents of detrended fluctuation analysis (DFA) (Peng et al., 1998; de Godoy, 2016; Gronwald and Hoos, 2020). To understand the cardiac output in relation to the pacing strategy, we also computed the cardiac cost (*CC*) (Billat et al., 2012), which is the ratio of the *BPM* and the running velocity.

#### Feature Computation

Following the extraction of parameters, we segmented the entire race into eight periods, each period corresponding to 12.5% of the race. The eight segments were selected to ensure the presence of (at least) one ROF value per segment. For every segment, we computed the median and interquartile range (IQR) for each biomechanical and physiological parameter. As the biomechanical and physiological metrics are highly subject-dependent, we normalized the values by dividing the median value of each segment by the median value of a reference segment. The race segment with the highest running speed was used as reference to normalize running biomechanics and the physiological parameters were normalized by considering the first segment as the “non-fatigued” state.

#### Computation of Association Measures

The goal of this analysis was assessing the association between the running gait parameters and the heart rate dynamics throughout the race. Based on existing results on the influence of acute fatigue on biomechanics, we selected five gait parameters—*t_c_*, *FSA*, *k*_*vert*_, *v*, and *a*_*AP*_ (Apte et al., 2021). We decided to include three physiological parameters—*BPM* as a classical metric, *SDNN* as the HRV metric, and the commonly used *DFA-α_1_* for HRC (Gronwald and Hoos, 2020; Gronwald et al., 2020b). We computed the Pearson correlation (Benesty et al., 2009) to characterize linear dependence and the distance correlation coefficient (Székely et al., 2007) to investigate the non-linear association. To obtain meaningful results from both the methods, the relevant time series must be synchronized, have the same sampling rate, and length. As a first step, each time series representing the entire race was segmented into 8 equal parts and outliers within moving windows of 10 samples (zero overlap) were replaced by the median of the window. Following this, within each segment, the mean values of the selected gait parameters (*t_c_*, *FSA*, *k*_*vert*_, *v*, and *a*_*AP*_) were computed on each 120 s rolling windows with an overlap of 10 s, ensuring the same pre-processing method as the physiological parameters. After ensuring similar sampling rate and length of time series, we then computed the linear and non-linear correlations for 104 segments, 8 for each of the 13 participants. This procedure was repeated for all 20 (=5×4) pairs of biomechanical and physiological parameters.

### Statistical Analysis

We conducted four statistical analyses (Figure 1C) to address the following research questions:

RQ1: How do biomechanical and physiological parameters evolve over the race progression?
RQ2: How do biomechanical and physiological parameters evolve over progression of perceived fatigue based on ROF values?
RQ3: At which level of perceived fatigue (ΔROF), are the biomechanical and physiological parameters significantly affected?
RQ4: What is the association between the gait and heart rate-derived parameters during endurance running and how does it change due to perceived fatigue?
RQ5: Are there noticeable differences between fast and slow runners in terms of fatigue progression and the evolution of different parameters?

Details of each analysis are provided below, with the statistical significance set at *p* ≤ 0.05. All analyses were performed with MATLAB R2020a (The MathWorks, United States).

#### RQ1 and RQ2: Influence of Fatigue

In order to investigate the effects of race progression on dependent variables (gait and ECG parameters), we applied the Friedman test, a non-parametric test to compare three or more repeated measurements, on segments S1 (begin), S5 (middle), and S8 (end) (Eisinga et al., 2017). The effect size was computed as:


(1) $$es_{F}=\frac{\chi^{2}}{n\left(k-1\right)}$$

Where *es*_*F*_ is the Kendall’s *W* test value, χ^2^ is the Friedman test statistic value, *n* is the sample size, and *k* is the number of measurements per subject (Tomczak and Tomczak, 2014). Kendall uses Cohen’s interpretation guidelines of 0.1 (small effect), 0.3 (moderate effect), and above 0.5 as a strong effect (Abdi, 2007). In addition, we computed pairwise comparisons (S1 vs. S5; S5 vs. S8 and S1 vs. S8) using the non-parametric Wilcoxon signed-rank test for paired observations. The effect size was defined as:


(2) $$es_{w}=\frac{Z}{\sqrt{N}}$$

where *Z* is the standardized *Z-score* and *N* is the total number of observations on which *Z* is based. To estimate the effects of fatigue based on the perceived fatigability, we compared segments with the lowest (L), medium (M), and highest (H) recorded ROF values. These fatigue levels were considered individually for each participant and pooled into three different groups (L, M, H) to overcome inter-subject variability in fatigue perception. When the same ROF value was observed on several segments, we computed the median parameter value for those segments. Further, we applied the Friedman and the Wilcoxon signed-rank test (L vs. M; M vs. H, and L vs. H) as previously explained. For both the pairwise comparisons, we did not use Bonferroni correction, since a small number of tests were performed (Armstrong, 2014).

#### RQ3: Onset of Change Based on Rating of Fatigue Values

The goal of this analysis was to investigate the onset of the biomechanical and physiological changes in response to perceived fatigability. To overcome inter-subject variability in ROF baseline values, we analyzed the ROF differences between segments (ΔROF), by subtracting each ROF value by that at the first segment (baseline). Since participants did not typically report a linear increase of fatigue, a resolution of ΔROF = 1 is inappropriate and would lead to multiple missing values. Consequently, we decided to create three states, by combining ΔROF 1 and 2, 3 and 4, and all values ≥ 5. When the same ΔROF values were obtained for several segments, we computed the median parameter value over those segments. Then, we applied the Friedman and Wilcoxon signed-rank tests, where each ΔROF > 0 was compared with ΔROF = 0 (i.e., 0 vs. [1–2]; 0 vs. [3–4]; and 0 vs. ≥ 5).

#### RQ4: Linear Mixed-Effects Models

A linear mixed-effects (LME) model was applied to investigate the influence of performances (i.e., fast vs. slow runners) on biomechanical and physiological metrics. We considered two groups of runners based on their performance, “fast” for five fastest runners (race time < 90 min) and “slow” for the five slowest runners (race time > 105 min). A 3-levels LME model was designed with the ΔROF, the performance, and the interaction between ΔROF and performance as the fixed effects (“ΔROF * performance” in Eq. 3). Then, a random effect (intercept and slope) on the subjects was defined [*“(ΔROF| subject)”* in Eq. 3]. As the LME model is robust to missing values, we did not group the ΔROF in three categories as explained in the previous section. The three levels correspond to the following models: level 1: within-subject model; level 2: within-group model (fast vs. slow); and level 3: between-group model. We provided the equation below as input to the “*fitlme”* Matlab function, with the “responder” corresponding to a biomechanical or physiological metric, and the “performance” corresponding to the fast and slow groups:


(3) responder∼ΔROF*performance+(ΔROF|subject)

Estimates of the model, *p*-value, and 95% confidence interval (CI) values of the fixed effects (intercept and slope) for both fast and slow groups were used to understand significant effects. Statistical significance was accepted for *p* ≤ 0.05 and if the range of the 95% CI did not include 0. In addition, the coefficient of determination (conditional $R_{c}^{2}$), was computed to assess the total variance explained by both fixed and random effects.

#### RQ5: Association Measures

For every pair of parameters, we computed 104 instances (8 segments × 13 subjects) of the linear Pearson correlation coefficient (*r*), the associated *p*-value (Cohen et al., 2009), and the nonlinear distance correlation coefficient (*dCor*) using the distance correlation (Székely et al., 2007). To explore the linear association within the 20 parameter pairs, we calculated the total number of segments with a significant linear correlation (*p* < 0.05). Further, to understand the strength of the correlations when all segments and subjects are pooled together, we computed the median (IQR) over the significant values of *r* and all *dcor* values.

## Results

All the thirteen participants (11 males, 2 females) were able to run until the end of the race (race time: 98.4 ± 12.3 min) without substantial walking bouts, and provided information about their ROF before race/after warm-up (3 ± 2) and after race (9 ± 1).

### RQ1 and RQ2: Influence of Fatigue

**FIGURE 2:**
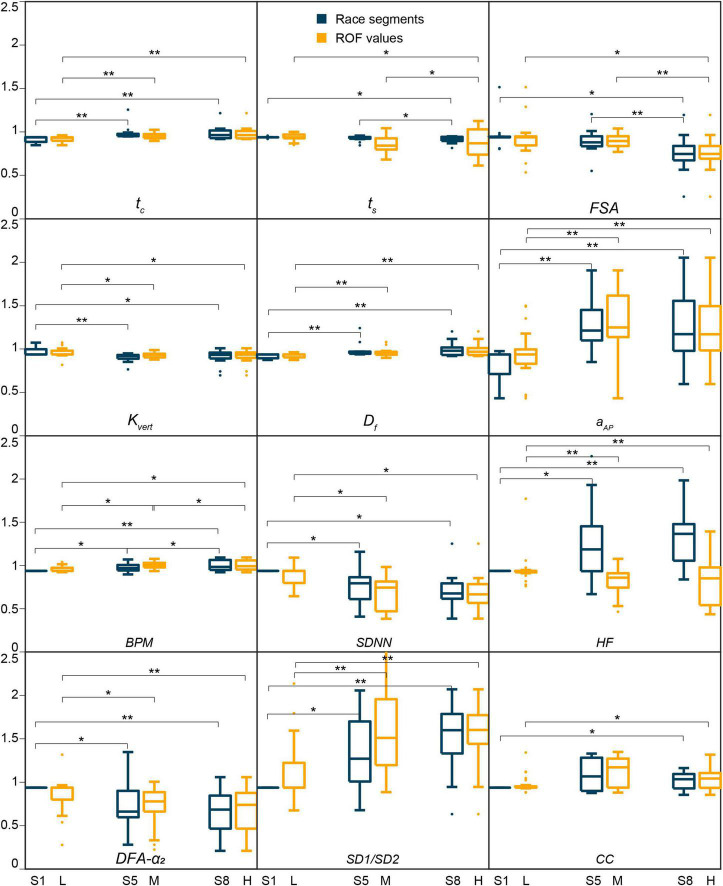
Parameters with a significant change with the race segments (in blue) and/or rating of fatigue (in yellow), with *p ∈ (0.01, 0.05) and **p ∈ (0.001, 0.01). S1, S5, and S8 represent the race segments 1, 5, and 8, and L, M, and H the low, medium, and high ROF values. Except *a*_*AP*_, all biomechanical parameters show substantially lower variability in trends than the physiological parameters.

**FIGURE 3:**
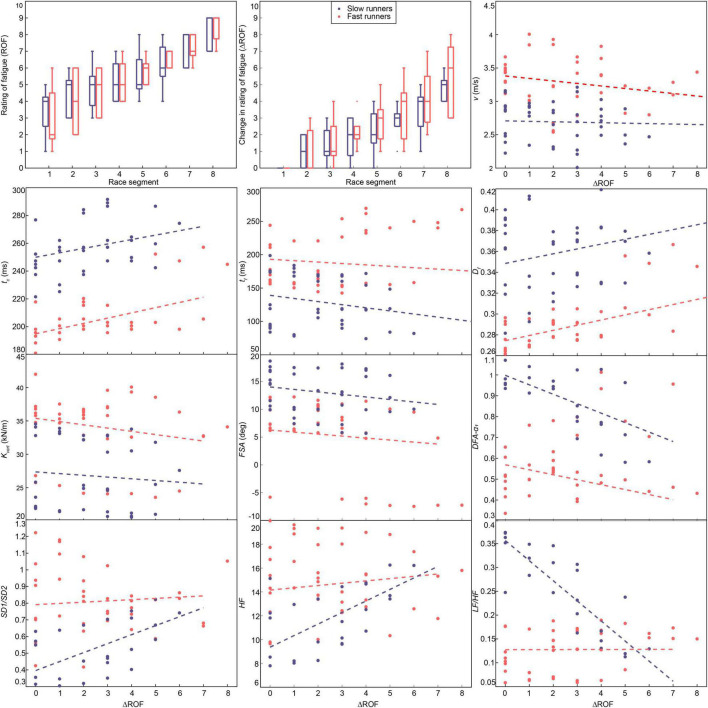
Change in the perceived fatigability with race progression and the results of the LME models for the response of the parameters, based on the “fast” and “slow” groups.

The influence of fatigue on biomechanical and physiological parameters, based on both race progression (RQ1) and ROF values (RQ2), is summarized in Table 1 and Figure 2. Actual values (median and IQR) of the parameters are reported in Supplementary Material. The increase in ROF and ΔROF scores throughout the race for all participants is presented in Figure 3 and shows an important inter-subject variability for the median ± IQR values at baseline [ROF(S1) = 4 ± 2]. Running a half-marathon affected spatiotemporal and heart rate metrics early in the race, mainly between segments 1 and 5. The *t_c_*, *D_f_*, and the *a*_*AP*_ values significantly increase during the race (*p* < 0.001, *es*_*F*_ > 0.5). The *FSA* (*p* < 0.001, *es*_*F*_ > 0.5) and the *k*_*vert*_ [*p* < 0.05, *es*_*F*_∈ (0.1, 0.3)] significantly decrease with high and low effect sizes respectively. Though the statistical tests reveal that the swing time and the peak swing vel. did not change significantly when comparing all three segments, significant differences are visible on the pairwise tests. The *t_c_*, ω_*s*_, the *k*_*vert*_, the *D_f_*, and the *a*_*AP*_ were altered at the beginning of the race as indicated by the S1| 5 significant results [*p* ∈ (0.001, 0.01)]. Only the *FSA* was altered during the second half of the race [*p* ∈ (0.001, 0.01)].

**TABLE 1:** Effect size results for the statistical analysis A1, A2, and A3 using Friedman (F) test and pairwise Wilcoxon signed-rank (WSR) test.

	RQ1 comparison across race segments				RQ2 comparison across ROF				RQ3 comparison across ΔROF			
	*F*-test (es_F_)	WSR test (es_W_)			*F*-test (es_F_)	WSR test (es_W_)			*F*-test (es_F_)	WSR test (es_W_)		
Parameter		S1| 5	S5| 8	S1| 8		L| M	M| H	L| H		0| [1,2]	0| [3,4]	0|≥ 5
*t*_*c*_	0.55***	0.62**	0.02	0.58**	0.41**	0.56**	0.34	0.57**	0.61***	0.58**	0.61**	0.61**
*t*_*f*_	0.17	0.34	0.16	0.28	0.06	0.20	0.17	0.28	0.10	0.31	0.32	0.32
*t*_*s*_	0.18	0.31	0.45*	0.39*	0.17	0.10	0.45*	0.43*	0.10	0.31	0.28	0.43*
*t*_*g*_	0.11	0.20	0.36	0.01	0.08	0.21	0.34	0.02	0.06	0.24	0.15	0.03
*Cad.*	0.11	0.20	0.36	0.02	0.08	0.21	0.34	0.02	0.04	0.22	0.14	0.03
*FSA*	0.57***	0.27	0.62**	0.43*	0.42**	0.20	0.53**	0.49*	0.39**	0.29	0.51	0.47*
*FEA*	0.02	0.28	0.12	0.21	0.00	0.06	0.17	0.20	0.03	0.17	0.25	0.19
ω_*s*_	0.17	0.43*	0.27	0.40*	0.25*	0.31	0.29	0.36	0.35**	0.35	0.57**	0.40*
*k*_*vert*_	0.26*	0.57**	0.08	0.43*	0.19	0.54*	0.09	0.43*	0.29*	0.58**	0.56**	0.47*
*D*_*f*_	0.54***	0.62**	0.13	0.58**	0.37**	0.55**	0.38	0.58**	0.69***	0.60**	0.61**	0.60**
*v*	0.08	0.25	0.32	0.21	0.03	0.08	0.05	0.10	0.06	0.10	0.16	0.10
*a*_*ML*_	0.09	0.32	0.23	0.24	0.01	0.20	0.06	0.13	0.05	0.15	0.19	0.24
*a*_*AP*_	0.79***	0.62**	0.09	0.62**	0.50**	0.58**	0.01	0.61**	0.44***	0.58**	0.61**	0.61**
*BPM*	0.38**	0.47*	0.47*	0.53**	0.32*	0.45*	0.39*	0.47*	0.24*	0.40*	0.45*	0.47*
*SDNN*	0.50**	0.47*	0.23	0.49*	0.35*	0.49*	0.13	0.46*	0.16	0.24	0.43*	0.43*
*RMSSD*	0.04	0.16	0.06	0.19	0.02	0.03	0.34	0.17	0.05	0.02	0.08	0.12
*DFA-α_1_*	0.01	0.14	0.13	0.05	0.00	0.02	0.09	0.03	0.02	0.13	0.05	0.03
*DFA-α_2_*	0.31*	0.43*	0.14	0.56**	0.35*	0.45*	0.16	0.54**	0.29*	0.39*	0.46*	0.51**
*SD1*	0.04	0.16	0.06	0.19	0.02	0.03	0.34	0.17	0.05	0.02	0.08	0.12
*SD2*	0.47**	0.47*	0.20	0.56**	0.33*	0.55**	0.10	0.54**	0.15	0.31	0.50*	0.53**
*SD1/SD2*	0.47**	0.49*	0.09	0.58**	0.41**	0.54**	0.14	0.54**	0.28*	0.43*	0.54**	0.53**
*pLF*	0.34*	0.40*	0.31	0.36	0.29*	0.42*	0.24	0.35	0.33**	0.29	0.45*	0.42*
*pHF*	0.34*	0.42*	0.34	0.43*	0.29*	0.43*	0.27	0.42*	0.33**	0.36	0.49*	0.45*
*LF*	0.12	0.17	0.25	0.21	0.09	0.08	0.23	0.16	0.09	0.10	0.19	0.12
*HF*	0.34*	0.43*	0.21	0.57**	0.58***	0.52**	0.24	0.62**	0.43***	0.40*	0.58**	0.57**
*LF/HF*	0.34*	0.39*	0.34	0.36	0.29*	0.40*	0.24	0.36	0.33**	0.29	0.45*	0.40*
*CC*	0.12	0.38	0.32	0.48*	0.03	0.30	0.02	0.40*	0.10	0.31	0.34	0.41*

S1, S5, and S8 indicate race segments 1, 5, and 8, whereas L, M, and H denote the low, median, and high ROF values. For significant results, effect size of (0.1, 0.3) was considered low, (0.3, 0.5) as medium, and > 0.5 as high for both WSR and F tests. The significance was set at p < 0.05, with * for p ∈ (0.01, 0.05), ** for p ∈ (0.001, 0.01), and *** for p < 0.001. Bold values correspond to significant results.

Regarding the heart rate metrics, the *BPM* significantly increased (*p* < 0.05), while the *SDNN* significantly decreased (*p* < 0.05). Despite a decreasing trend, non-significant changes were observed for *RMSSD* during the race. The *pLF*, *LH/HF* ratio, and *SD2* significantly decreased during running (*p* < 0.05). On the contrary, *HF*, *pHF*, and *SD1/SD2* increased with race progression (*p* < 0.05) (Table 1 and Figure 2). Surprisingly, the *DFA-α_1_* did not significantly change during the race (*p* > 0.05), while the *DFA-α_2_* decreased significantly (*p* < 0.05). The significant alterations of the above-mentioned physiological parameters were observed during the first half of the race and remain until the end, as indicated by significant pairwise comparison between segments 1 and 5, and segments 1 and 8 (Table 1).

Comparisons across ROF values showed similar trends for the spatiotemporal and physiological parameters as those based on race progression (Table 1 and Figure 2). Remarkably, we observed slightly higher effect sizes across race segments than across ROF values for all the parameters with significant changes, with *HF* showing an inverse tendency.

### RQ3: Onset of Change

Table 1 also provides the evolution of the biomechanical and physiological parameters across fatigue scores, where ΔROF values are pooled in four states (i.e., 0, [1,2], [3,4], and ≥ 5). Unsurprisingly, parameters showing significant alterations in RQ1 and RQ2 analysis, also present significant changes in RQ3. However, these results provide a deeper understanding of the onset of change based on the perceived fatigability. The spatiotemporal biomechanical parameters, *t_c_* (*p* < 0.001), *D_f_* (*p* < 0.001),

*k*_*vert*_ (*p* < 0.05) and *a*_*AP*_ (*p* < 0.001) show significant changes at all fatigue states including ΔROF 1 and 2 [*p* ∈ (0.001, 0.01), *es*_*W*_ > 0.5]. Then, a significant decrease of peak swing vel. appears at moderate fatigue states [ΔROF = [3–4], *p* ∈ (0.001, 0.01)]. Finally, *FSA* and *t_s_* values became significantly lower only at high fatigue scores (ΔROF > 5).

Concerning the HRV metrics, the time-domain *BPM*, the frequency-domain *HF*, and the non-linear metrics *DFA-α_2_* and *SD1/SD2* ratio changed significantly at all fatigue states; first with medium effect sizes [*p* < 0.05, *es*_*W*_∈ (0.3, 0.5)] for low ΔROF, then high effect sizes at higher fatigue states (ΔROF > 5). The *SDNN*, *SD2*, *pLF*, *pHF*, and *LF/HF* ratio were affected at medium and high fatigue scores. Finally, the cardiac cost significantly increased at high perceived exertion, when ΔROF ≥ 5 [*p* < 0.05, *es*_*W*_∈ (0.3, 0.5)].

### RQ4: Investigation According to Performance

The results of the influence of performances (i.e., fast vs. slow runners) on biomechanical and physiological metrics, based on the‘ LME model, are presented in Figure 3. Only a subset of metrics showing significant differences between groups on fixed-effects, intercept or slope, are presented. Interestingly, the spatiotemporal biomechanical parameters showed significant differences in the intercept values between fast and slow runners, while the slopes were similar (Figure 3). Compared to fast runners, the slower group presented a higher *t_c_*, *D_f_*, and *FSA*, and lower *k*_*vert*_ (Figure 3) throughout the race.

For physiological parameters, the frequency-domain (*pLF*, *pHF*, *LF*, *HF*, and *LF/HF* ratio) and the non-linear (*SD1/SD2* ratio, *DFA-α_1_*) metrics showed significant fixed-effects (intercepts and slopes) between groups. Figure 3 indicates that *DFA-α_1_* was higher for slow runners at baseline and proceeded to decrease for both groups. It is worth mentioning that two runners in the fast group were considered outliers in the LME model as having high *DFA-α_1_* values (∼ 1) at the end of the race, leading to high residuals. Interestingly, *SD1/SD2* and *LF/HF* ratios demonstrated converging trends (Figure 3), as slow runners started with lower *SD1/SD2* ratio values compared to fast runners and vice-versa for *LF/HF* ratio. Whereas those ratios seem relatively stable for the fast group throughout the race, a significant increase and decrease are visible for *SD1/SD2* and *LF/HF* ratios, respectively. The estimate, *p*-values, 95% confidence interval (CI), and conditional $R_{c}^{2}$of the fixed effects (intercept and slope) for both fast and slow groups are reported in Supplementary Material.

### RQ5: Association Measures

**FIGURE 4:**
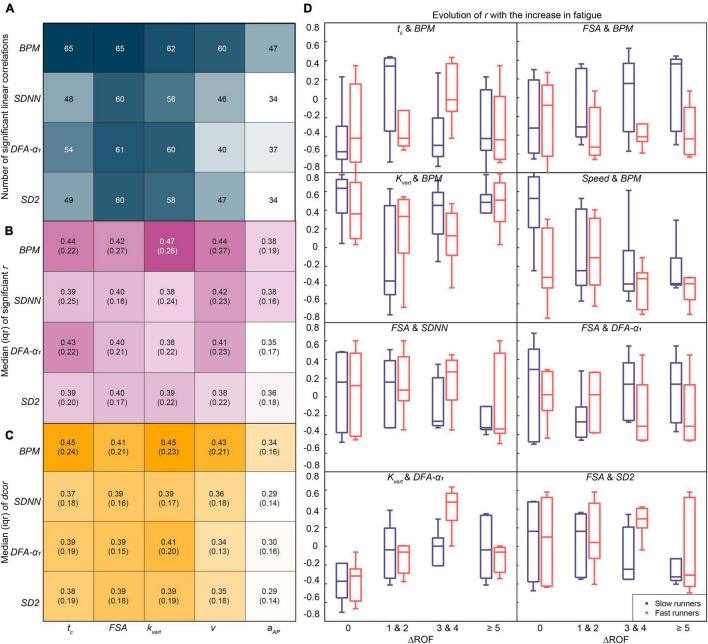
Analysis of the linear and non-linear similarity metrics for the selected gait and physiological parameters. **(A)** Number of parameter pairs with significant linear correlations out of a total of 104 pairs, **(B)** median and interquartile range (IQR) of the significant linear Pearson correlation coefficient (r) across subjects and segments, and **(C)** median (IQR) of the non-linear distance correlation coefficient (dcor). **(D)** Investigation of linear similarity metric (r) for parameter pairs with at least 60 significant correlations, based on the performance of the participants (slow and fast runners).

Based on the literature on the influence of acute fatigue (Apte et al., 2021), we selected five gait parameters—*t_c_*, *FSA*, *k*_*vert*_, *v*, and *a*_*AP*_, and three physiological parameters—*BPM*, *SDNN*, *DFA-α_1_* for association analysis. In addition, we decided to include *SD2 a posteriori*, because our current findings demonstrated strong trends for the long-range HRC variables (*DFA-α_2_*, and *SD2*). *FSA* and *BPM* typically showed the highest number of significant linear correlations (Figure 4A) with other physiological and biomechanical parameters, respectively; *a*_*AP*_ showed the lowest. Consistent with this, the magnitude of the *r* and the *dcor* was relatively higher for *t_c_*| *BPM*, *k*_*vert*_| *BPM*, *v*| *BPM*, *FSA*| *BPM*, *FSA*| *DFA-α_1_*, and *FSA*| *SD2* pairs. *a*_*AP*_ showed the lowest strength of association with any of the physiological parameters. Figure 4D presents the evolution of the *r* value for the four ΔROF states, with *FSA*| *BPM*, *k*_*vert*_| *BPM*, *FSA*| *DFA-α_1_*, and *FSA*| *SDNN* showing different trends for slow and fast groups. *k*_*vert*_| *DFA-α_1_*, *FSA*| *SDNN*, *FSA| BPM*, and *FSA| SD2* show a change of the correlation pattern with the increase in perceived fatigability.

## Discussion

The goal of the present study was to measure concurrently and continuously the response of the biomechanical, physiological, and psychological parameters to acute fatigue during a half-marathon run. The influence of fatigue on biomechanical and physiological parameters, based on race progression and ROF values, is discussed in section “Influence of Fatigue” (RQ1 and RQ2, respectively). The onset of changes based on ΔROF values is considered in section “Onset of Fatigue” (RQ3). Finally, the comparison of fast with slow runners (RQ4) and the linear and non-linear association metrics for the selected gait and physiological parameters (RQ5) are explained in section “Differences Between Slow and Fast Runners” and “Association Between Biomechanical and Physiological Parameters,” respectively.

### Influence of Fatigue

Concerning the biomechanical parameters, our analysis confirms previous results (Apte et al., 2021; Meyer et al., 2021), showing a stable gait cycle time, an increase in contact time and duty factor, as well as decreases in pitch angle, swing time and vertical stiffness (Figure 2). The alteration in running biomechanics observed in the present study results from strategies to compensate for neuromuscular fatigue (Apte et al., 2021). Vertical stiffness represents the global response of spring-mass model to acute fatigue, thus rendering it crucial to the understanding of biomechanical changes (Morin et al., 2005). Decreased vertical stiffness indicates an increase in the vertical motion of the COM and/or a decrease in the peak vertical GRF. The decrease in vertical stiffness is consistent with the observations in shorter time trials (800 m) but not for a longer mountainous ultramarathon race (330 km) distance (Degache et al., 2016; Girard et al., 2017). However, these comparisons must remain anecdotal due to the difference in running conditions, intensities and in methods for stiffness estimation. In addition to lower body biomechanical changes, we observed a significant increase in the trunk anteroposterior acceleration, most likely linked with a fatigue of the lower back postural muscles. Except the *FSA*, the above-mentioned biomechanical alterations appeared during the first half of the race, and maintained throughout the race (Table 1). These findings are consistent with previous studies demonstrating that a large amount of muscle activation impairments is obtained early on a self-paced exercise (Azevedo et al., 2019). The widely recognized critical point associated to fatigue in marathon race, known as “hitting the wall,” and characterized by a late-race slowdown (Buman et al., 2008), was not observed when performing group statistics in our study. The fact that a majority of our participants were not “hitting the wall,” shown by a reasonably stable running speed, might explain why we do not observe additional significant alterations of the biomechanical parameters during the second part (between S5 and S8).

Interestingly, the heart rate metrics are also affected early in the race, mainly between segments 1 and 5. As expected, some time-domain HRV metrics, expressed via *SDNN*, decreases from the beginning of the exercise (Gronwald et al., 2020a). Regarding the frequency-domain metrics, in agreement with Casties et al. (2006), our results demonstrate a decrease in *LF/HF* ratio during running. As *LF* does not change significantly, the *LF/HF* ratio decrease is mainly resulting from an increase in *HF* power. The frequency band of *HF* corresponds to heart rate variations related to the respiratory cycle, and a shift in respiration rate and volume can critically change *HF* power (Blain et al., 2005; Shaffer and Ginsberg, 2017). Thus, the observed increase in *HF* (decrease in *LF/HF* ratio) during the race is most likely linked to an increase in breathing rate.

Gronwald et al. (2020b) suggests using *DFA-α_1_* as a proxy for the complex regulation of the central and autonomous nervous system in response to different exercise intensities. However, those *DFA-α_1_* changes were mainly analyzed in-laboratory setting during incremental tests. Only one study reported a significant decrease of HR fractal properties during a marathon race (Gronwald et al., 2021). In our results, short-term scaling exponents *DFA-α_1_* was not significantly lower at the end of the race compared to the baseline when all participants are combined (Table 1). However, a significant difference between fast and slow runners is observed (Figure 3) as well as a significant decrease of *DFA-α_1_* in both groups. Despite lack of investigation with *DFA-α_2_* in literature, we considered relevant to report its results as it showed significant decrease throughout the race. Regarding the Poincare plot, the non-significant change in *SD1* during the race, accompanied with a significant decrease in *SD2*, suggests that sympathetic activation results in progressive reduction in the long-term oscillation of HR (Tulppo et al., 1996). The nonlinear measures of HR dynamics provide useful information during exercise, especially the metrics reflecting long-term fluctuations of HR (*SD2* and *DFA-α_2_*), that are not easily detected by linear measures of HRV. These changes of HR dynamics from strongly correlated to uncorrelated or anti-correlated indicates a behavior dependent on exercise intensity, likely induced by a vagal withdrawal and/or sympathetic activation (Platisa and Gal, 2008).

Neuromuscular fatigue, which in part explains the running pattern alterations, accompanied by changes in metabolic and physiological demands, contributes to the increase of perceived fatigue. We notice only few differences for physiological and biomechanical parameter trends based on ROF (low, medium, high), compared to race progression. The main difference resides in smaller effect sizes for A2 statistical analysis, which might be explained by high inter-subject variability in perceived fatigue. This was caused by the pooling in A2 statistics, which led to the parameters at highest ROF of highly fatigued runners being pooled with the parameters at highest ROF of moderately fatigued runners. Thus, it seems relevant to compute ΔROF as a fatigue score for assessing acute fatigue. Furthermore, we did not necessarily find a linear increase of ROF, in contrast to earlier findings during in-laboratory incremental tests (Gronwald et al., 2018). This underlines the importance of measuring perceived fatigue during a running event that involves pacing strategies.

### Onset of Fatigue

We observed biomechanical and physiological alterations at different fatigue states compared to the baseline value. The contact time, duty factor, stiffness, and *a*_*SG*_ show significant alterations at all fatigue states. Then, peak swing vel., *FSA*, and swing time significantly change at higher fatigue scores. These early changes in running pattern are most likely linked with a drop in muscle contractile function at the beginning of the race, as previously demonstrated (Azevedo et al., 2019). Moreover, once the biomechanical parameters start changing, the participants find it difficult to recover the deteriorating running technique.

Concurrent physiological adaptations are observed with a significant increase in *BPM*, *HF*, and *SD1*/*SD2* ratio, and a significant decrease in *DFA-α_2_* at all fatigue states (from ΔROF [1–2] to ΔROF ≥ 5). Later, *SDNN*, *SD2*, *pHF*, *pLF*, *LF/HF* ratio, and *CC* are affected as well by acute fatigue. The decrease in HRV and HRC, accompanied with an increase in *HF* and *pHF*, support previous findings demonstrating the persistence of respiration sinus arrythmia (RSA) at high workloads (Blain et al., 2005; Prigent et al., 2021), as it is the main source of HRV at high exercise intensities.

Furthermore, our results demonstrate that the gait parameters are affected by a lower increase in fatigue compare to the physiological parameters, which are affected at higher fatigue levels (Table 1). Interestingly, some biomechanical parameters are affected from the first sensation of fatigue (ΔROF [1,2]), suggesting a correlation between perceived fatigue and neuromuscular impairments; these impairments are known as the underlying mechanism responsible for running technique alteration. This observation is in line with other studies suggesting that peripheral muscle fatigue would be the constantly regulated variable (Calbet, 2006), with a continuous sensory feedback coming from working muscles to the central nervous system (Esteve-Lanao et al., 2008). Neuromuscular fatigue seems to be the dominant mechanism influencing perceived fatigue during the initial portion of the run. Later, the feedback from the fatiguing cardiorespiratory system might also increase the perception of effort (Bergstrom et al., 2015). Finally, as demonstrated by an increase in cardiac cost at high fatigue perception, we can argue that additional motor units are needed to produce the same overall muscle efficiency, which results in higher physiological/metabolic costs (Kounalakis et al., 2008; Marcora et al., 2008). These neuromuscular and cardiorespiratory afferent sensory feedbacks, among others, are subconsciously processed in the brain, resulting in an unpleasant sensation of fatigue, which directly influences the pacing strategy. This difference in pacing strategy was clearly visible in both the fast and slow runner groups.

### Differences Between Slow and Fast Runners

The sample size in our study is too low to conclude any statistical results for between-group comparison of fast and slow runners. However, the clear trends for each group could be relevant for a future between-group study design. Concerning the biomechanical parameters, the rate of alterations throughout the race are comparable between these two groups, the main differences reside in the intercept values (Figure 3). Interestingly, the athletes considered as well-trained in our study (fast group) present more stable physiological parameters (*DFA-α_1_*, *SD1*/*SD2* ratio, *HF*, and *LF*/*HF* ratio) during the race than less-trained participants (Figure 3). As all participants, fast and slow included, reported an increase in ROF scores during the race, the evidence from the previous section indicating a correlation between perceived fatigue and neuromuscular impairments seems to be confirmed.

Moreover, our results suggest that less trained subjects might feel additional fatigue signs due to lower cardiorespiratory capacities and thus adopt a cautious pacing compared to fast runners, making sure they can finish the race (Noakes et al., 2005). In contrast, the fast runners seem to push harder from the beginning as seen by a slight decrease in speed in the second part of the race, whereas the slow group adopt a stable and lower running speed.

This leads to a progressive alteration of HRV parameters for the slow group, indicated by progressive changes of the heart rate metrics during the race (i.e., *DFA-α_1_*, *SD1*/*SD2* ratio, *HF*, and *LF*/*HF* ratio), converging toward the values obtained for fast runners. Well-trained runners might sustain high metabolic cost for a prolonged period, explaining the quick and sustained drop in the HRV and HRC metrics. Low *DFA-α_1_* values (∼0.6) are already measured for fast runners in the 10 first minutes of the race; and maintained throughout the race, whereas the slow group showed a progressive reduction in HRC.

### Association Between Biomechanical and Physiological Parameters

Our results demonstrated a substantially higher number of correlations with biomechanical parameters for heart rate, compared to HRV and HRC. Due to the increase in muscle fatigue, a higher number of motor units should be recruited to maintain the same muscle force, leading to a need for an increased neural drive (Girard et al., 2012). The circulatory strain is further increased due to dehydration and rise in the core body temperature (Kenefick et al., 2012), leading to a progressive increase in the heart rate throughout the race. Indeed, heart rate (*BPM*) is the only parameter that shows a significant difference between segments 1, 5, and 8, and for low, medium, and high ROF values. The measured relative change in R-R intervals decreases with an increase in heart rate, even if the variability of heart rate is the same (Sacha, 2014). This can explain the relatively lower number of correlations for HRV and HRC with biomechanical parameters, owing to a reduction in their measured change.

Another interesting result is the high number of correlations for the *FSA*. This could be linked to the increase in *FSA*, which is directly related to the leg muscle fatigue and the adaptation of running kinematics to acute fatigue (Apte et al., 2021; Meyer et al., 2021). Medio-lateral trunk acceleration (*a*_*AP*_) showed the lowest number of significant linear correlations and lowest value of distance correlation for all physiological parameters. Thus, *a*_*AP*_ could be considered as an independent response variable for acute fatigue, as it shows a significant increase but is not related to physiological parameters. Trunk muscles are slow twitch muscles, thus engendering lower cost for maintaining posture (Bramble and Lieberman, 2004), as compared to the cost for maintaining similar speed with the leg muscles. Concurrent recording of biomechanical and physiological parameters enabled an analysis of their association and its evolution with perceived fatigability. Within the framework of the new emerging field of Network Physiology and Complex Systems Science, investigating how physiological systems and subsystems coordinate and interact, has shown promise in understanding diverse exercise-related phenomena such as sports performance, fatigue, or sport injuries (Balagué et al., 2020). Using wearable sensors, our study demonstrates the feasibility of following this approach during an in-field prolonged running event. Our investigation can be extended further by the analysis of raw ECG and IMU signals from the trunk sensor using the causality, stability, modularity, frequency-domain approaches, etc. However, care must be taken to ensure that the ECG signal is free of movement artifacts and the different sources of signals are correctly synchronized. Based on our results, we recommend further controlled analysis and hypothesis testing to understand the reasons behind the higher correlations for the eight pairs of parameters presented in Figure 4.

### Limitations and Recommendations

The estimation of *FSA* can be rendered less accurate for participants with a forefoot strike (Falbriard et al., 2020), which was the case with one participant in the fast group. Concerning the heart rate metrics, the presence of artifacts might influence the frequency and non-linear indexes. Despite correction methods and 5% artifact threshold for data exclusion, substantial bias in the extracted metrics could happen (Giles and Draper, 2018). Giles and Draper (2018), recommended using near artifact free method when analyzing heart rate variability during high intensity exercise. An additional limitation is the unclear physiological interpretation of non-linear metrics (*DFA-α_1_*, *DFA-α_2_*, *SD1/SD2* ratio). Gronwald et al., suggested using *DFA-α_1_* as a “global parameter” for the whole system and as a proxy for the complex regulation of the central and autonomous nervous system (Gronwald et al., 2020b). However, the exact factors influencing *DFA-α_1_* are still unclear, such as the possible influence of breathing and cardiorespiratory coupling. Assessment of respiration can also provide information about cardiorespiratory coordination, which has been shown to be sensitive to the short-term and long-term effects of exercise (Garcia-Retortillo et al., 2017). Consequently, we suggest measuring breathing patterns in future studies aiming to assess fatigue.

The number of subjects in the fast/slow groups is low and the overall sample is limited to 13 subjects. Background data about the participants, such their VO2max values, sleep quality, stress, and emotional health can improve the interpretation of the results. In addition, improving the resolution of the collection of ROF samples can enable a finer analysis of the evolution of perceived fatigability and its influence on the biomechanical and physiological parameters. Finally, the perceived fatigability can be assessed more holistically by also including the measurement of the valence, arousal, flow state, and action crisis (Venhorst et al., 2018). While this additional measurement was not feasible for us during the race, a pre/post assessment could provide a more complete understanding of the affective, sensory, and cognitive processes.

## Conclusion

This work is one of the first to concurrently and continuously measure the response of biomechanical, physiological, and psychological parameters to acute fatigue during a half-marathon run. The biomechanical parameters presented a significant alteration even with a small change in perceived fatigue, whereas the heart rate dynamics alter at higher fatigue levels. When analyzed as two groups using a LMEs model, the slower runners showed a higher change in heart rate dynamics throughout the race than the faster runners; whereas both groups developed similar trends for the gait parameters. When tested for linear and non-linear correlations, heart rate presented the highest association with biomechanical parameters, while the *a*_*SG*_ showed the lowest association with heart rate dynamics. These results indicate the ability of faster runners to better perceive their physiological limits and hint toward a higher sensitivity of perceived fatigue to changes in the running gait. This study highlights measurable influences of acute fatigue, which can be studied only through concurrent measurement of biomechanical, physiological, and psychological facets of running in real-world conditions. It may serve as a springboard for the design of studies that measure the association of biomechanical and physiological parameters and its evolution with acute fatigue. Utilization of such wearable sensor setups can further allow a more personalized approach to fatigue analysis and thereby enable an improved customization of training programs.

## Data Availability Statement

The raw data supporting the conclusions of this article will be made available by the authors to a qualified researcher, without undue reservation.

## Ethics Statement

The studies involving human participants were reviewed and approved by EPFL human research ethics committee (HREC 039-2018). The patients/participants provided their written informed consent to participate in this study.

## Author Contributions

GP and SA performed the data collection, data analysis, and wrote the first draft of the manuscript. All authors contributed to the study design, discussion of the obtained data and results, and the final manuscript. All authors reviewed the final manuscript and assumed responsibility for the information presented therein.

## Conflict of Interest

The authors declare that the research was conducted in the absence of any commercial or financial relationships that could be construed as a potential conflict of interest. The reviewer GM declared a shared affiliation with several of the authors CB and VG to the handling editor at the time of review.

## Publisher’s Note

All claims expressed in this article are solely those of the authors and do not necessarily represent those of their affiliated organizations, or those of the publisher, the editors and the reviewers. Any product that may be evaluated in this article, or claim that may be made by its manufacturer, is not guaranteed or endorsed by the publisher.

## References

[B1] Abdi H. (2007). “The Kendall rank correlation coefficient,” in *Encyclopedia of Measurement and Statistics*, ed. Salkind N. J. (Thousand Oaks, CA: Sage), 508–510.

[B2] Alexander R. M. (1991). Energy-saving mechanisms in walking and running. *J. Exp. Biol.* 160 55–69. 10.1242/JEB.160.1.55 1960518

[B3] Alfuth M. Rosenbaum D. (2011). Long distance running and acute effects on plantar foot sensitivity and plantar foot loading. *Neurosci. Lett.* 503 58–62. 10.1016/j.neulet.2011.08.010 21871535

[B4] Apte S. Meyer F. Gremeaux V. Dadashi F. Aminian K. (2020). A sensor fusion approach to the estimation of instantaneous velocity using single wearable sensor during sprint. *Front. Bioeng. Biotechnol.* 8:838. 10.3389/fbioe.2020.00838 33014992 PMC7461787

[B5] Apte S. Prigent G. Stöggl T. Martínez A. Snyder C. Gremeaux-Bader V. (2021). Biomechanical response of the lower extremity to running-induced acute fatigue: a systematic review. *Front. Physiol.* 12:646042. 10.3389/fphys.2021.646042 34512370 PMC8430259

[B6] Armstrong R. A. (2014). When to use the Bonferroni correction. *Ophthalmic Physiol. Opt.* 34 502–508. 10.1111/opo.12131 24697967

[B7] Azevedo R. de A. Cruz R. Couto P. Silva-Cavalcante M. D. Boari D. (2019). Characterization of performance fatigability during a self-paced exercise. *J. Appl. Physiol.* 127 838–846. 10.1152/japplphysiol.00090.2019 31318614

[B8] Balagué N. Hristovski R. Almarcha M. Garcia-Retortillo S. Ivanov P. C. (2020). Network physiology of exercise: vision and perspectives. *Front. Physiol.* 11:1607. 10.3389/FPHYS.2020.611550/BIBTEX PMC7759565 33362584

[B9] Benesty J. Chen J. Huang Y. Cohen I. (2009). Pearson correlation coefficient. *Springer Top. Signal. Process.* 2 1–4. 10.1007/978-3-642-00296-0_5

[B10] Benson L. C. Clermont C. A. Bošnjak E. Ferber R. (2018). The use of wearable devices for walking and running gait analysis outside of the lab: a systematic review. *Gait Posture* 63 124–138. 10.1016/j.gaitpost.2018.04.047 29730488

[B11] Bergstrom H. C. Housh T. J. Cochrane K. C. Jenkins N. D. M. Zuniga J. M. Buckner S. L. (2015). Factors underlying the perception of effort during constant heart rate running above and below the critical heart rate. *Eur. J. Appl. Physiol.* 115 2231–2241. 10.1007/S00421-015-3204-Y 26108674

[B12] Billat V. L. Palacin F. Correa M. Pycke J.-R. (2019). Pacing strategy affects the sub-elite marathoner’s cardiac drift and performance. *Front. Psychol.* 10:3026. 10.3389/fpsyg.2019.03026 32140116 PMC7043260

[B13] Billat V. L. Palacin F. Correa M. Pycke J.-R. (2020). Pacing strategy affects the sub-elite marathoner’s cardiac drift and performance. *Front. Psychol.* 10:3026. 10.3389/FPSYG.2019.03026 32140116 PMC7043260

[B14] Billat V. L. Petot H. Landrain M. Meilland R. Koralsztein J. P. Mille-Hamard L. (2012). Cardiac output and performance during a marathon race in middle-aged recreational runners. *Sci. World J.* 2012:810859. 10.1100/2012/810859 22645458 PMC3356747

[B15] Billman G. E. Huikuri H. V. Sacha J. Trimmel K. (2015). An introduction to heart rate variability: methodological considerations and clinical applications. *Front. Physiol.* 6:810859. 10.3389/FPHYS.2015.00055 25762937 PMC4340167

[B16] Blain G. Meste O. Bermon S. (2005). Influences of breathing patterns on respiratory sinus arrhythmia in humans during exercise. *Am. J. Physiol. Heart Circ. Physiol.* 288 H887–H895. 10.1152/ajpheart.00767.2004 15388504

[B17] Blickhan R. (1989). The spring-mass model for running and hopping. *J. Biomech.* 22 1217–1227. 10.1016/0021-9290(89)90224-8 2625422

[B18] Borg G. A. (1982). Psychophysical bases of perceived exertion. *Med. Sci. Sports Exerc.* 14 377–381. 7154893

[B19] Bourdon P. C. Cardinale M. Murray A. Gastin P. Kellmann M. Varley M. C. (2017). Monitoring athlete training loads: consensus statement. *Int. J. Sports Physiol. Perform.* 12 S2–S161. 10.1123/IJSPP.2017-0208 28463642

[B20] Bramble D. M. Lieberman D. E. (2004). Endurance running and the evolution of Homo. *Nature* 432 345–352. 10.1038/nature03052 15549097

[B21] Buckley C. O’Reilly M. A. Whelan D. Farrell A. V. Clark L. Longo V. (2017). “Binary classification of running fatigue using a single inertial measurement unit,” in *Proceedings of the 2017 IEEE 14th Int Conf Wearable Implant Body Sens Networks, BSN 2017*, Eindhoven. 10.1109/BSN.2017.7936040

[B22] Buman M. P. Brewer B. W. Cornelius A. E. Van Raalte J. L. Petitpas A. J. (2008). Hitting the wall in the marathon: phenomenological characteristics and associations with expectancy, gender, and running history. *Psychol. Sport Exerc.* 9 177–190. 10.1016/j.psychsport.2007.03.003

[B23] Calbet J. A. L. (2006). The rate of fatigue accumulation as a sensed variable. *J. Physiol.* 575 688. 10.1113/JPHYSIOL.2006.116087 16840512 PMC1995686

[B24] Camomilla V. Bergamini E. Fantozzi S. Vannozzi G. (2018). Trends supporting the in-field use of wearable inertial sensors for sport performance evaluation: a systematic review. *Sensors* 18:873. 10.3390/s18030873 29543747 PMC5877384

[B25] Caruso M. Sabatini A. M. Knaflitz M. Gazzoni M. Della Croce U. Cereatti A. (2019). “Accuracy of the orientation estimate obtained using four sensor fusion filters applied to recordings of magneto-inertial sensors moving at three rotation rates,” in *Proceedings of the 2019 41st Annual International Conference of the IEEE Engineering in Medicine and Biology Society (EMBC)*, Berlin, 2053–2058. 10.1109/EMBC.2019.8857655 31946305

[B26] Casties J.-F. Mottet D. Le Gallais D. (2006). Non-linear analyses of heart rate variability during heavy exercise and recovery in cyclists. *Int. J. Sports Med.* 27 780–785. 10.1055/s-2005-872968 16586334

[B27] Ceyssens L. Vanelderen R. Barton C. Malliaras P. Dingenen B. (2019). Biomechanical risk factors associated with running-related injuries: a systematic review. *Sport. Med.* 49 1095–1115. 10.1007/S40279-019-01110-Z 31028658

[B28] Clermont C. A. Benson L. C. Edwards W. B. Hettinga B. A. Ferber R. (2019). New considerations for wearable technology data: changes in running biomechanics during a marathon. *J. Appl. Biomech.* 35 1–9. 10.1123/jab.2018-0453 31629343

[B29] Cohen I. Huang Y. Chen J. Benesty J. (2009). *Noise Reduction in Speech Processing*, Vol. 2. Berlin: Springer. 10.1007/978-3-642-00296-0

[B30] Cottin F. Medigue C. Lopes P. Lepretre P.-M. Heubert R. Billat V. (2007). Ventilatory thresholds assessment from heart rate variability during an incremental exhaustive running test. *Int. J. Sports Med.* 28 287–294. 10.1055/s-2006-924355 17024637

[B31] de Godoy M. F. (2016). Nonlinear analysis of heart rate variability: a comprehensive review. *J. Cardiol. Ther.* 3 528–533. 10.17554/J.ISSN.2309-6861.2016.03.101-4

[B32] Degache F. Morin J.-B. Oehen L. Guex K. Giardini G. Schena F. (2016). Running mechanics during the world’s most challenging mountain Ultramarathon. *Int. J. Sports Physiol. Perform.* 11 608–614. 10.1123/ijspp.2015-0238 26457730

[B33] Eisinga R. Heskes T. Pelzer B. Te Grotenhuis M. (2017). Exact p-values for pairwise comparison of Friedman rank sums, with application to comparing classifiers. *BMC Bioinformatics* 18:68. 10.1186/s12859-017-1486-2 28122501 PMC5267387

[B34] Enoka R. M. Duchateau J. (2016). Translating fatigue to human performance. *Med. Sci. Sports Exerc.* 48 2228–2238. 10.1249/MSS.0000000000000929 27015386 PMC5035715

[B35] Eskofier B. Kugler P. Melzer D. Kuehner P. (2012). “Embedded classification of the perceived fatigue state of runners: towards a body sensor network for assessing the fatigue state during running,” in *Proceedings of the 2012 9th International Conference on Wearable and Implantable Body Sensor Networks*, London, 113–117. 10.1109/BSN.2012.4

[B36] Esteve-Lanao J. Lucia A. deKoning J. J. Foster C. (2008). How do humans control physiological strain during strenuous endurance exercise? *PLoS One* 3:e2943. 10.1371/JOURNAL.PONE.0002943 18698405 PMC2491903

[B37] Falbriard M. Meyer F. Mariani B. Millet G. P. Aminian K. (2018). Accurate estimation of running temporal parameters using foot-worn inertial sensors. *Front. Physiol.* 9:610. 10.3389/fphys.2018.00610 29946263 PMC6005819

[B38] Falbriard M. Meyer F. Mariani B. Millet G. P. Aminian K. (2020). Drift-free foot orientation estimation in running using wearable IMU. *Front. Bioeng. Biotechnol.* 8:65. 10.3389/fbioe.2020.00065 32117943 PMC7031162

[B39] Fredette A. Roy J.-S. Perreault K. Dupuis F. Napier C. Esculier J.-F. (2021). The association between running injuries and training parameters: a systematic review. *J. Athl. Train.* 10.4085/1062-6050-0195.21 [Epub online ahead of print] 34478518 PMC9528699

[B40] Garcia-Retortillo S. Javierre C. Hristovski R. Ventura J. L. Balagué N. (2017). Cardiorespiratory coordination in repeated maximal exercise. *Front. Physiol.* 8:387. 10.3389/fphys.2017.00387 28638349 PMC5461287

[B41] Giles D. A. Draper N. (2018). Heart rate variability during exercise: a comparison of artefact correction methods. *J. Strength Cond. Res.* 32 726–735. 10.1519/JSC.0000000000001800 29466273

[B42] Girard O. Millet G. P. Micallef J. P. (2017). Mechanical alterations during 800-m self-paced track running. *Int. J. Sports Med.* 38 314–321. 10.1055/S-0042-121262/ID/R5779-0032 28249345

[B43] Girard O. Millet G. P. Micallef J. P. Racinais S. (2012). Alteration in neuromuscular function after a 5 km running time trial. *Eur. J. Appl. Physiol.* 112 2323–2330. 10.1007/s00421-011-2205-8 22012541

[B44] Gronwald T. Hoos O. (2020). Correlation properties of heart rate variability during endurance exercise: a systematic review. *Ann. Noninvasive Electrocardiol.* 25:e12697. 10.1111/anec.12697 31498541 PMC7358842

[B45] Gronwald T. Hoos O. Hottenrott K. (2020a). Influence of performance level of male runners on non-linear dynamics of heart rate variability during a 10Km race. *Int. J. Perform. Anal. Sport* 20 569–583. 10.1080/24748668.2020.1764746

[B46] Gronwald T. Rogers B. Hoos O. (2020b). Fractal correlation properties of heart rate variability: a new biomarker for intensity distribution in endurance exercise and training prescription? *Front. Physiol.* 11:1152. 10.3389/fphys.2020.550572 33071812 PMC7531235

[B47] Gronwald T. Hoos O. Ludyga S. Hottenrott K. (2018). Non-linear dynamics of heart rate variability during incremental cycling exercise. *Res. Sports Med.* 27 88–98. 10.1080/15438627.2018.1502182 30040499

[B48] Gronwald T. Rogers B. Hottenrott L. Hoos O. Hottenrott K. (2021). Correlation properties of heart rate variability during a marathon race in recreational runners: potential biomarker of complex regulation during endurance exercise. *J. Sport. Sci. Med.* 20 557–563. 10.52082/jssm.2021.557 PMC8488837 35321146

[B49] Hautala A. J. Mäkikallio T. H. Seppänen T. Huikuri H. V. Tulppo M. P. (2003). Short-term correlation properties of R-R interval dynamics at different exercise intensity levels. *Clin. Physiol. Funct. Imaging* 23 215–223. 10.1046/j.1475-097X.2003.00499.x 12914561

[B50] Jayasekera S. Hensel E. Robinson R. (2021). Feasibility assessment of wearable respiratory monitors for ambulatory inhalation topography. *Int. J. Environ. Res. Public Health* 18:2990. 10.3390/ijerph18062990 33799472 PMC8000968

[B51] Kenefick R. W. Cheuvront S. N. Sawka M. N. (2012). Thermoregulatory function during the marathon. *Sport Med.* 37 312–315. 10.2165/00007256-200737040-00010 17465596

[B52] Knicker A. J. Renshaw I. Oldham A. R. H. Cairns S. P. (2011). Interactive processes link the multiple symptoms of fatigue in sport competition. *Sport Med.* 41 307–328. 10.2165/11586070-000000000-00000 21425889

[B53] Kounalakis S. N. Nassis G. P. Koskolou M. D. Geladas N. D. (2008). The role of active muscle mass on exercise-induced cardiovascular drift. *J. Sports Sci. Med.* 7:395. 24149908 PMC3761905

[B54] Kumar D. K. Arjunan S. P. Aliahmad B. (2017). *Fractals: Applications in Biological Signalling and Image Processing.* Boca Raton, FL: CRC Press, 10.1201/9781315165868

[B55] Marcora S. M. Bosio A. de Morree H. M. (2008). Locomotor muscle fatigue increases cardiorespiratory responses and reduces performance during intense cycling exercise independently from metabolic stress. *Am. J. Physiol. Regul. Integr. Comp. Physiol.* 294 874–883. 10.1152/AJPREGU.00678.2007 18184760

[B56] Meyer F. Falbriard M. Mariani B. Aminian K. Millet G. P. (2021). Continuous analysis of marathon running using inertial sensors: hitting two walls? *Int. J. Sports Med.* 42 1182–1190. 10.1055/a-1432-2336 33975367

[B57] Micklewright D. St Clair Gibson A. Gladwell V. Al Salman A. (2017). Development and validity of the rating-of-fatigue scale. *Sport Med.* 47 2375–2393. 10.1007/s40279-017-0711-5 28283993 PMC5633636

[B58] Millet G. Y. (2011). Can neuromuscular fatigue explain running strategies and performance in ultra-marathons? *Sport Med.* 41 489–506. 10.2165/11588760-000000000-00000 21615190

[B59] Morin J. B. Dalleau G. Kyröläinen H. Jeannin T. Belli A. (2005). A simple method for measuring stiffness during running. *J. Appl. Biomech.* 21 167–180. 10.1123/jab.21.2.167 16082017

[B60] Noakes T. D. St Clair Gibson A. Lambert E. V. (2005). From catastrophe to complexity: a novel model of integrative central neural regulation of effort and fatigue during exercise in humans: summary and conclusions. *Br. J. Sports Med.* 39 120–124. 10.1136/bjsm.2003.010330 15665213 PMC1725112

[B61] Novacheck T. F. (1998). The biomechanics of running. *Gait Posture* 7 77–95. 10.1016/S0966-6362(97)00038-6 10200378

[B62] Op De Beéck T. Meert W. Schütte K. (2018). “Fatigue prediction in outdoor runners via machine learning and sensor fusion,”.in *Proceedings of the 24th ACM SIGKDD International Conference on Knowledge Discovery & Data Mining*, Vol. 57 New York, NY, 1184–1192. 10.1016/0003-4975(94)91354-4

[B63] Pageaux B. Lepers R. (2016). Fatigue induced by physical and mental exertion increases perception of effort and impairs subsequent endurance performance. *Front. Physiol.* 7:587. 10.3389/fphys.2016.00587 27965592 PMC5126404

[B64] Paquette M. R. Napier C. Willy R. W. Stellingwerff T. (2020). Moving beyond weekly “distance”: optimizing quantification of training load in runners. *J. Orthop. Sport Phys. Ther.* 50 564–569. 10.2519/jospt.2020.9533 32741325

[B65] Peng C. K. Havlin S. Stanley H. E. Goldberger A. L. (1998). Quantification of scaling exponents and crossover phenomena in nonstationary heartbeat time series. Chaos an interdiscip. *J. Nonlinear Sci.* 5:82. 10.1063/1.166141 11538314

[B66] Platisa M. M. Gal V. (2008). Correlation properties of heartbeat dynamics. *Eur. Biophys. J.* 37 1247–1252. 10.1007/s00249-007-0254-z 18210101

[B67] Prigent G. Aminian K. Rodrigues T. Vesin J.-M. Millet G. P. Falbriard M. (2021). Indirect estimation of breathing rate from heart rate monitoring system during running. *Sensors* 21:5651. 10.3390/S21165651 34451093 PMC8402314

[B68] Rincon Soler A. I. Silva L. E. V. Fazan R. Jr. Murta L. O. Jr. (2018). The impact of artifact correction methods of RR series on heart rate variability parameters. *J. Appl. Physiol.* 124 646–652. 10.1152/japplphysiol.00927.2016 28935830

[B69] Robusto C. C. (1957). The cosine-haversine formula. *Am. Math. Mon.* 64 38–40. 10.2307/2309088

[B70] Rogers B. Giles D. Draper N. Mourot L. Gronwald T. (2021). Influence of artefact correction and recording device type on the practical application of a non-linear heart rate variability biomarker for aerobic threshold determination. *Sensors* 21:821. 10.3390/s21030821 33530473 PMC7865269

[B71] Rothschild C. E. (2012). Primitive running: a survey analysis of runners’ interest, participation, and implementation. *J. Strength Cond. Res.* 26 2021–2026. 10.1519/JSC.0b013e31823a3c54 21997446

[B72] Ruder M. Jamison S. T. Tenforde A. Mulloy F. Davis I. S. (2019). Relationship of foot strike pattern and landing impacts during a marathon. *Med. Sci. Sports Exerc.* 51 2073–2079. 10.1249/MSS.0000000000002032 31525171

[B73] Sacha J. (2014). Interaction between heart rate and heart rate variability. *Ann. Noninvasive Electrocardiol.* 19 207–216. 10.1111/ANEC.12148 24602150 PMC6932101

[B74] Schmitt L. Regnard J. Millet G. P. (2015). Monitoring fatigue status with HRV measures in elite athletes: an avenue beyond RMSSD? *Front. Physiol.* 6:343. 10.3389/fphys.2015.00343 26635629 PMC4652221

[B75] Shaffer F. Ginsberg J. P. (2017). An overview of heart rate variability metrics and norms. *Front. Public Health* 5:258. 10.3389/fpubh.2017.00258 29034226 PMC5624990

[B76] Strohrmann C. Harms H. Kappeler-Setz C. Troster G. (2012). Monitoring kinematic changes with fatigue in running using body-worn sensors. *IEEE Trans. Inf. Technol. Biomed.* 16 983–990. 22677321 10.1109/TITB.2012.2201950

[B77] Székely G. J. Rizzo M. L. Bakirov N. K. (2007). Measuring and testing dependence by correlation of distances. *Ann. Stat.* 35 2769–2794. 10.1214/009053607000000505

[B78] Tarvainen M. P. Niskanen J.-P. Lipponen J. A. Ranta-Aho P. O. Karjalainen P. A. (2014). Kubios HRV–heart rate variability analysis software. *Comput. Methods Programs Biomed.* 113 210–220. 10.1016/j.cmpb.2013.07.024 24054542

[B79] Task Force of the European Society of Cardiology the North American Society of Pacing Electrophysiology (1996). Heart rate variability. *Circulation* 93 1043–1065. 10.1161/01.CIR.93.5.1043 8598068

[B80] Thorpe R. T. Atkinson G. Drust B. Gregson W. (2017). Monitoring fatigue status in elite team-sport athletes: implications for practice. *Int. J. Sports Physiol. Perform.* 12 27–34. 10.1123/ijspp.2016-0434 28095065

[B81] Tomczak M. Tomczak E. (2014). The need to report effect size estimates revisited. an overview of some recommended measures of effect size. *Trends Sport Sci.* 21 19–25.

[B82] Tulppo M. P. Makikallio T. H. Takala T. E. Seppanen T. Huikuri H. V. (1996). Quantitative beat-to-beat analysis of heart rate dynamics during exercise. *Am. J. Physiol.* 271(1 Pt 2) H244–H252. 8760181 10.1152/ajpheart.1996.271.1.H244

[B83] Vargas N. T. Marino F. (2014). A neuroinflammatory model for acute fatigue during exercise. *Sport Med.* 44 1479–1487. 10.1007/s40279-014-0232-4 25164464

[B84] Venhorst A. Micklewright D. Noakes T. D. (2018). Perceived fatigability: utility of a three-dimensional dynamical systems framework to better understand the psychophysiological regulation of goal-directed exercise behaviour. *Sport Med.* 48 2479–2495. 10.1007/S40279-018-0986-1/FIGURES/2 30238409

[B85] Verschueren J. Tassignon B. Pauw K. Proost M. Teugels A. van Cutsem J. (2020). Does acute fatigue negatively affect intrinsic risk factors of the lower extremity injury risk profile? A systematic and critical review. *Sport Med.* 50 767–784. 10.1007/s40279-019-01235-1 31782066

[B86] Yazdani S. Fallet S. Vesin J.-M. (2018). A novel short-term event extraction algorithm for biomedical signals. *IEEE Trans. Biomed. Eng.* 65 754–762. 10.1109/TBME.2017.2718179 28644795

